# The Microstructure and Properties of Hard Anodic Oxide Coatings on 5754 Aluminium Alloy Modified with Al_2_O_3_, PTFE and CaCO_3_ Nanoparticles

**DOI:** 10.3390/ma19020378

**Published:** 2026-01-17

**Authors:** Anna Kozik, Marek Nowak, Kamila Limanówka, Anna Góral

**Affiliations:** 1 Institute of Metallurgy and Materials Science, Polish Academy of Sciences, 25 Reymonta Street, 30-059 Kraków, Poland; 2 Łukasiewicz Research Network-Institute of Non-Ferrous Metals, 19 Piłsudskiego Street, 32-050 Skawina, Poland; marek.nowak@imn.lukasiewicz-gov.pl (M.N.); kamila.limanowka@imn.lukasiewicz.gov.pl (K.L.)

**Keywords:** hard anodic oxide coating, anodizing, Al_2_O_3_, CaCO_3_, PTFE nanoparticles, abrasion resistance

## Abstract

Hard anodic oxide coatings on aluminium have long been used to enhance surface functionality. However, increasing industrial demands are driving the need for coatings with superior hardness, wear resistance, corrosion resistance and self-lubricating properties. Due to their porous structure, anodic oxide coatings can be modified by incorporating various nanoparticles. The properties of the modified coatings depend on both the type of nanoparticles used and the method employed to incorporate them. In this study, anodic oxide coatings were produced using direct and duplex methods on a semi-industrial scale to enable process control and potential industrial implementation. The coatings were modified with hard (Al_2_O_3_) and soft (CaCO_3_, PTFE) nanoparticles in order to customise their functional properties. Their microstructure and chemical composition were characterised by SEM and TEM. Their microhardness, abrasion resistance and electrochemical behaviour were also evaluated. Among the tested production methods and methods for modifying nanoparticles, the duplex process incorporating Al_2_O_3_ particles proved to be the most promising. Its optimisation resulted in coatings with a microhardness of 430 HV0.05 and a mass loss of 9.4 mg after the Taber abrasion test, demonstrating the potential of this approach for industrial applications.

## 1. Introduction

Aluminium alloys have been widely used in the manufacture of mechanical friction components due to their favourable thermal conductivity, light weight and minimal operating noise. However, their properties, such as relatively low hardness, high friction coefficient and poor lubricity, limit their practical application. Therefore, to improve these properties, anodic oxide coatings were formed on aluminium alloys.

Anodic oxide coatings and the mechanisms of their formation have been well-known for many years [1,2,3]. These coatings exhibit a porous structure composed of densely arranged hexagonal cells, each containing a central pore. Due to this unique morphology, the oxide layer demonstrates an ability to absorb, enabling its modification by incorporating hard particles or solid lubricants.

The type of modifier used determines the way in which the surface layer of aluminium alloys can be shaped. To reduce friction in sliding pairs, solid lubricant particles such as graphite [4], molybdenum disulfide [5,6] and tungsten disulfide [7] are used as modifiers. Polymers are used to impart self-lubricating properties to the coating and reduce the friction coefficient. The most commonly used material is polytetrafluoroethylene (PTFE) [1,4,8,9] while other examples include perfluoroether (PEPE) and decyltrichlorosilane (OTS) [10]. Polymer-modified coatings are already available on an industrial scale under the trade name TUFRAM [11]. To achieve enhanced their tribological and mechanical properties, the coatings are modified with hard particles such as aluminium oxide (Al_2_O_3_) [3,12], silicon carbide (SiC) [13], silicon nitride (Si_3_N_4_) [14], titanium dioxide (TiO_2_) [15] and zirconium dioxide (ZrO_2_) [16].

Several methods exist for introducing modifying particles into the porous structure of an anodic oxide coating. These methods can generally be divided into two categories.

In the first approach, the particles are added directly to the electrolyte solution used for anodic oxidation and are incorporated into the porous structure during the anodising process itself [17]. The second approach is the so-called duplex method, in which the anodic oxide coating is produced first and subsequently modified. In this method, the particles form a separate layer primarily on the surface of the anodic oxide coating. Particle modification can be carried out using ultrasonic impregnation [18], vacuum impregnation [19], electrophoretic deposition [4] or by simply immersing the anodised component in a particle suspension [20]. Other methods also exist, such as ion implantation, cathodic sputtering and vacuum arc deposition; however, these techniques are considerably more expensive [14].

Researchers have enhanced the tribological properties of anodic oxide coatings through surface modifications. For instance, Chen et al. [17] incorporated Al_2_O_3_ and PTFE particles into alumina nanopores using a direct method with various current techniques. Zhang et al. [21] employed a duplex method, first producing the coating and then adding PTFE particles via electrophoresis or ultrasonic impregnation. These composite coatings demonstrated reduced friction coefficients (0.15–0.26) and improved wear resistance compared to unmodified coatings.

The present work aimed to determine the most effective processing route for producing anodic oxide coatings with enhanced mechanical and tribological properties. The coatings were produced using common direct and rarely used duplex methods on a semi-industrial scale to ensure better control of the process parameters and enable the technology to be transferred to industrial applications more easily. A new approach utilising both direct and duplex methods, as well as hard and soft particles as modifiers, was adopted to tailor the coatings’ functional properties. Hard particles, such as Al_2_O_3_, were used to improve microhardness, wear resistance and corrosion resistance [22], whereas soft particles, including CaCO_3_ and PTFE, were used to enhance tribological properties. Calcium carbonate, a common ingredient in lubricants, increases wear resistance, load-bearing capacity and pressure endurance [23], while polytetrafluoroethylene (PTFE) provides self-lubricating properties and significantly reduces the friction coefficient [9].

The presented studies focus on hard anodic oxide coatings intended for aluminium machine components and production line elements, such as pump pistons, rotors operating in a tobacco dust environment and linear guides used in transport systems. These applications require high resistance to mechanical and tribological wear. The development of coatings with improved properties aims to extend the service life of these components, reduce the frequency of regeneration or replacement and lower costs and downtime in industrial processes.

## 2. Materials and Methods

EN-AW 5754 aluminium alloy, used as an anode, was cut into 100 × 100 × 3 mm. The chemical composition was determined using optical emission spectroscopy with an ARL 4460 spectrometer (Thermo Fisher Scientific, Waltham, MA, USA), and the measurement results are presented in (Table 1).

Before anodization, the aluminium alloy was degreased in acetone, etched in 200 g/L sodium hydroxide (NaOH, Chempur, Piekary Śląskie, Poland) solution for 5 min, followed by brightening in nitric acid (HNO_3_, VWR, Gdańsk, Poland) solution for 2 min. An aluminium sheet was used as a cathode.

Aqueous suspensions of Al_2_O_3_ (Bimotech, Wrocław, Poland), CaCO_3_ and PTFE (Nanochemazone, Leduc, AB, Canada) nanoparticles were prepared in the presence and absence of selected surfactants in order to evaluate their influence on colloidal stability. According to the manufacturers’ declarations, the average particle size (D_50_) was 20 nm for both CaCO_3_ and α-Al_2_O_3_. Meanwhile, PTFE particles exhibited a D_50_ in the range of 30–50 nm. The zeta potential of each suspension was measured to determine the effectiveness of the surfactants in preventing particle agglomeration. Measurements were performed using a ZETASIZER NANO ZS90 (Malvern Instruments, Malvern, UK) equipped with U-shaped DTS1061 measurement cells. Three surfactants supplied by Thermo Fisher Scientific (Waltham, MA, USA) were selected for this study: sodium dodecylbenzenesulfonate (SDBS), imide of sulfobenzoic acid (LSA) and sodium 2-ethylhexyl sulfosuccinate (DSS). A constant volume of surfactants (1 g/L) and nanopowders (0.2 g/L) was used. The pH, a key factor affecting the zeta potential was adjusted in 1-unit increments (2–6). Measurements were conducted at 25 °C (standard zeta potential measurement temperature) and 0 °C (the temperature of the galvanic bath).

Nanoparticle-modified hard anodic oxide coatings were produced using direct and duplex methods. In the direct method, coatings were anodized in a 0.3 M oxalic acid (Chempur, Piekary Śląskie, Poland) bath with 1 g/L DSS surfactant and 5 g/L nanopowders in two stages (30 min at 2 A/dm^2^, followed by 60 min at 4 A/dm^2^). According to previous studies [24], the use of oxalic acid solution and variable current parameters enables the formation of pores with a diameter of approximately 50 nm, which is suitable for subsequent modification. In the duplex method, coatings were prepared as in the direct one, and then subjected to pore widening in phosphoric acid and ultrasonic impregnation for 10 min (cycle 0.7 s on, 0.4 s off, 50% amplitude) in a nanopowder suspension containing 5 g/L nanopowders and 1 g/L DSS surfactant. The fabrication process of Al_2_O_3_-modified coatings produced using the duplex method was modified by increasing the nanoparticle concentration in the suspension to 10 and 20 g/L and extending the impregnation time to 20 and 30 min (for the 20 g/L concentration).

Given the focus of this study being on tribological properties and the need to maintain high wear resistance, the coatings produced in this research did not undergo a sealing treatment, even after incorporating nanoparticles into the anodic layer.

The coatings’ microstructure and cross-section analysis were conducted using a HR-SEM (field emission gun, FEG) equipped with an Energy-Dispersive X-ray Spectroscopy (EDS, IN-SPECT F50, FEI Company, Hillsboro, OR, USA). To enhance conductivity, a 10 nm carbon layer was deposited via magnetron sputtering, and samples were mounted using silver adhesive. The chemical composition analysis of the coatings in micro-areas was performed using EDS. Vickers microhardness was measured on the coating surface using a Qness CHD 60 Master Plus tester (Qness GmbH, Salzburg, Austria), averaging five measurements. The test was conducted in accordance with ISO 6507-1:2023 [25]. The measurements were performed using a test load of HV 0.05 with a test force duration of $14_{-4}^{+1}$ s. The abrasion test was carried out using a Taber Abraser Model 5155 tester (Taber Industries, North Tonawanda, NY, USA), CS-17 abrasive wheels and a load of 1000 g, over 10,000 cycles, under controlled environmental conditions (50 ± 5% humidity, 23 ± 2 °C), according to ISO 10074:2017 standard [23].

Electrochemical tests using AUTOLAB PGSTAT 302 and GPES 4.9 software (Metrohm Autolab B.V., Utrecht, The Netherlands) utilised potentiodynamic methods (1 mV/s) after 1 h OCP stabilisation. Anodic oxide coatings were tested with a platinum auxiliary electrode and Ag/AgCl reference electrode, in 3.5% NaCl, at 25 °C.

## 3. Results

### 3.1. Nanopowders

**Figure 1:**
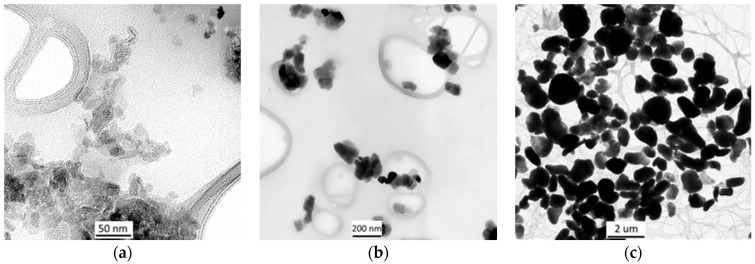
TEM morphology of nanopowders Al_2_O_3_ (**a**); CaCO_3_ (**b**); and PTFE (**c**).

The TEM morphology of the Si_3_N_4_ particles used in this study is presented in Figure 1.

The nanopowders differ in both particle shape and their tendency to agglomerate. The Al_2_O_3_ particles are relatively well dispersed, although they form irregular structures. In contrast, the CaCO_3_ and PTFE powders exhibit a pronounced tendency to create agglomerates with irregular morphology, particularly in the case of PTFE, where the formation of dense clusters makes it difficult to clearly distinguish individual particles.

### 3.2. Zeta Potential

The zeta potential represents the electric potential at the slipping plane of dispersed particles and is a key parameter governing electrostatic interactions in colloidal systems. For nanoparticle suspensions, sufficiently high absolute zeta potential values prevent particle agglomeration due to electrostatic repulsion. Surfactants modify the surface charge of nanoparticles through adsorption, thereby altering the electrical double layer and improving suspension stability.

**Figure 2:**
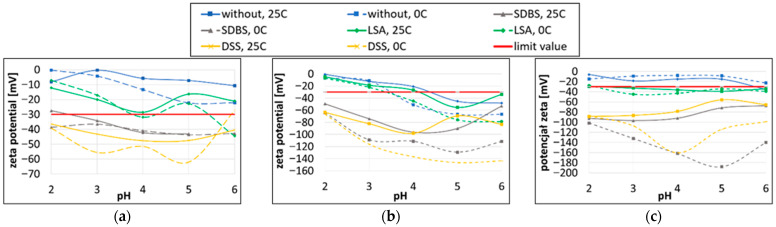
Zeta potential as a function of pH for suspension containing nanoparticles (**a**) Al_2_O_3_; (**b**) CaCO_3_; and (**c**) PTFE.

The graphs in Figure 2 show the average zeta potential values obtained for each type of nanoparticles as a function of the pH, temperature and surfactant used. An absolute zeta potential value of 30 mV is adopted as the criterion for the electrostatic stability of the suspension and is marked on the graphs with a red line. The measurements were carried out at two temperatures: 25 °C—the standard temperature for zeta potential measurements—and 0 °C—the operating temperature of the galvanic bath at Łukasiewicz-IMN.

The zeta potential measurements for the suspension containing Al_2_O_3_ particles (Figure 2a) revealed that the suspension without the addition of surfactants exhibited an absolute zeta potential value below the electrostatic stability threshold. In contrast, the addition of the surfactants SDBS and DSS resulted in a pronounced increase in the absolute zeta potential value. Among the investigated surfactants, DSS demonstrated the highest stabilising efficiency, yielding absolute zeta potential values exceeding 60 mV over a broad pH range, at both 0 °C and 20 °C. For the soft particles CaCO_3_ (Figure 2b) and PTFE (Figure 2c), the surfactants DSS and SDBS also exhibited the most pronounced effect in terms of enhancing the absolute zeta potential, ensuring electrostatic stabilisation across the entire investigated range of pH and temperature.

Table 2 presents the zeta potential values measured at 0 °C, as this temperature corresponds to the operating conditions of the galvanic bath during coating production. This is particularly important for coatings fabricated using the direct method, where the stability of the nanoparticle suspension directly affects the deposition process and the quality of the resulting coating. Stability is achieved when the absolute value of the zeta potential is above 30 mV. The analysis shows that DSS and SDBS effectively increased the zeta potential, improving stability and reducing agglomeration of the nanoparticles.

Among the surfactants tested, sodium sulfosuccinate (DSS) resulted in the most significant reduction in the absolute zeta potential and therefore was selected for further investigations in the coating production process.

### 3.3. Properties of the Coatings Produced by the Direct and Duplex Methods with a Nanoparticle Concentration of 5 g/L

#### 3.3.1. The Morphology of the Coatings

**Figure 3:**
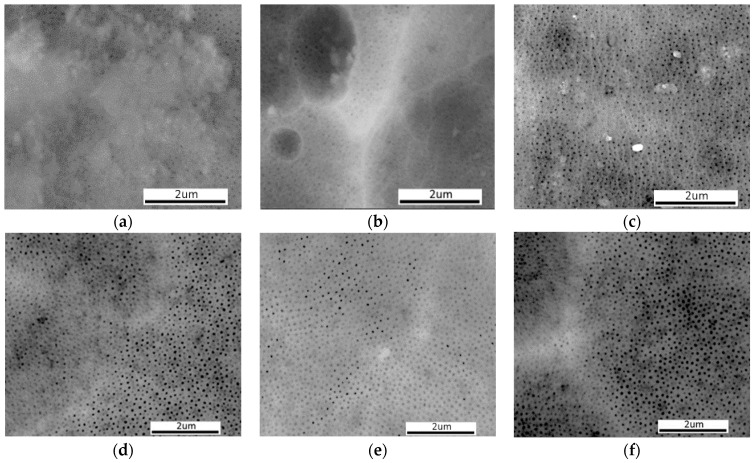
SEM-SE morphology of the coatings produced by the direct method: (**a**) Al_2_O_3_, (**b**) PTFE and (**c**) CaCO_3_; and by the duplex method: (**d**) Al_2_O_3_, (**e**) PTFE and (**f**) CaCO_3_.

The surface morphologies of the coatings produced by the direct method are illustrated in Figure 3a–c while those produced by the duplex method are shown in Figure 3d–f. SEM examinations of both sets of coatings reveal noticeable areas on all surfaces where the pores of the coatings are closed, which appear as lighter regions.

**Figure 4:**
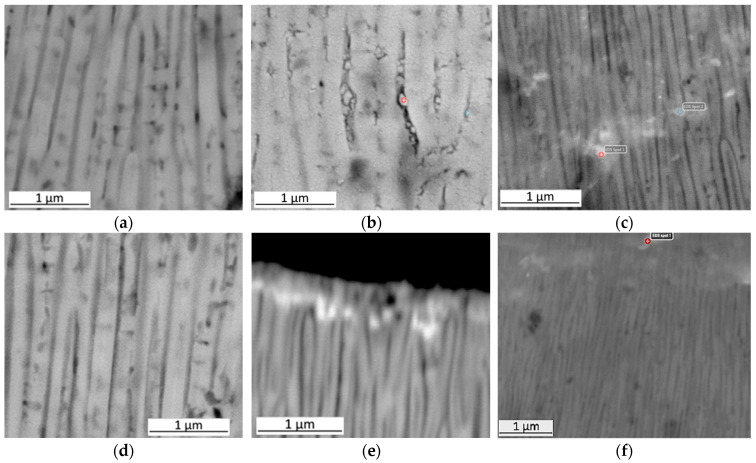
SEM–SE cross-sections of the coatings produced by the direct method: (**a**) Al_2_O_3_, (**b**) PTFE and (**c**) CaCO_3_; and by the duplex method: (**d**) Al_2_O_3_, (**e**) PTFE and (**f**) CaCO_3_. The red points in the figure indicate the locations of the modifying nanoparticles, as confirmed by EDS analysis.

In these places, the analysis of the chemical composition by the EDS method, both on the surface and cross-sections (Figure 4), showed the presence of aluminium (Al), magnesium (Mg) and oxygen (O) in the case of coatings modified with Al_2_O_3_ nanoparticles, small amounts of fluorine (F) in the coatings modified with PTFE nanoparticles, and calcium (Ca) in the coatings modified with CaCO_3_ nanoparticles—both in the case of coatings produced by the direct and duplex method.

#### 3.3.2. Microhardness, Abrasion Resistance and Corrosion Resistance

**Figure 5:**
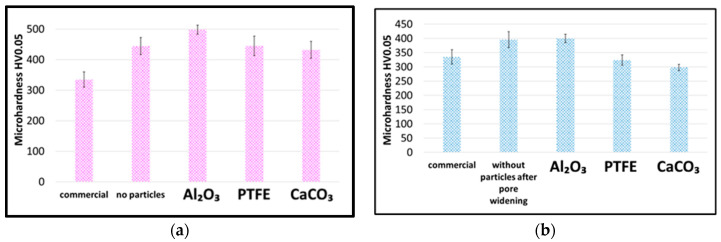
Vicker’s microhardness of coatings produced using the direct method (**a**) and duplex method (**b**).

**Figure 6:**
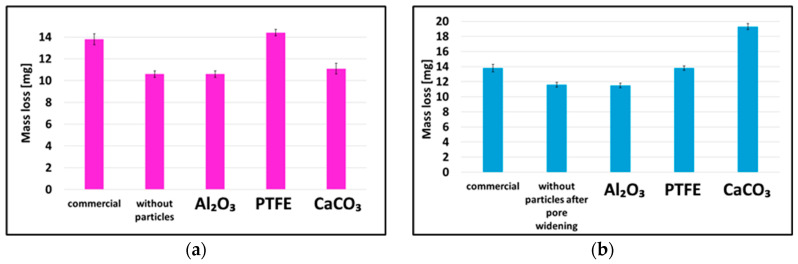
Abrasion resistance (mass loss after Taber test) of coatings produced by direct method (**a**) and duplex method (**b**).

The results of the microhardness (Figure 5) and abrasion resistance (Figure 6) measurements compare the properties of coatings produced by the direct and duplex methods to the commercial coatings produced at Łukasiewicz-IMN and to unmodified coatings. Hard Al_2_O_3_ particles increased microhardness, reaching 498 HV0.05 in the direct method (Figure 4a), while soft CaCO_3_ and PTFE particles reduced it, with CaCO_3_ yielding the lowest value (298 HV0.05). PTFE-modified coatings had a microhardness value ranging from 250 to 370 HV, depending on process parameters [4].

Abrasion resistance tests showed no significant improvement with Al_2_O_3_ particles, while CaCO_3_ and PTFE increased mass loss (Figure 6).

**Figure 7:**
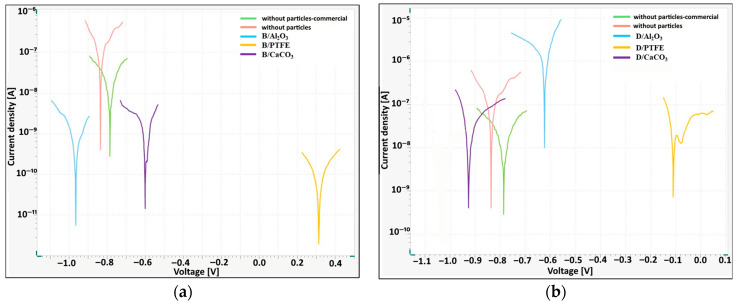
Polarisation curves of coatings produced by (**a**) direct method; (**b**) duplex method.

To determine the corrosion resistance of the coatings, electrochemical tests were carried out using the potentiodynamic method in a 3.5% NaCl solution. The results are presented in Figure 7 and Table 3 for both production methods.

In the potentiodynamic tests, a shift in the corrosion potential (E_corr_) toward more positive values indicates a more thermodynamically stable surface behaviour, a lower corrosion current density (j_corr_) corresponds to a reduced corrosion rate and a higher polarisation resistance (Rp) reflects the improved protective properties and enhanced corrosion resistance of the coating. The decrease in the corrosion current density for all the coatings modified with nanoparticles during the direct method indicates their better corrosion resistance compared to the unmodified anodic oxide coating. In the case of the duplex method, the incorporation of PTFE nanoparticles also led to a shift in the polarisation curves toward more positive potential values, demonstrating a corrosion behaviour comparable to that observed for coatings produced by the direct method. Despite the fact that the Al_2_O_3_ nanoparticle-modified coating produced by the duplex method exhibited a relatively high corrosion current density and a low polarisation resistance, it showed a more positive corrosion potential compared to the unmodified coatings. The improvement in the corrosion resistance of the nanoparticle-modified coatings is due to their presence in the coating structure. The presence of nanoparticles leads to an increase in the surface density and thus the corrosion resistance of the produced layer [26].

#### 3.3.3. Summary of Section 3.3

The analysis aimed to identify the optimal type of modifying nanoparticles, production methods and process parameters for hard anodic oxide coatings with enhanced mechanical properties and improved abrasive wear resistance compared to the commercial coatings produced at Łukasiewicz-IMN, while meeting the requirements of ISO 10074 and the Qualanod standards. The results showed that soft particles (PTFE and CaCO_3_) increased mass loss after the Taber test regardless of the production method, whereas the incorporation of Al_2_O_3_ nanoparticles enhanced coatings’ microhardness while maintaining a wear resistance comparable to the reference coating. Consequently, Al_2_O_3_ nanoparticles and the duplex production method were selected for further optimisation. This choice was additionally supported by the technological advantages of the duplex method, particularly the lower suspension volume required compared to the highly exothermic direct method, which necessitates significantly larger bath volumes and higher amounts of costly nanoparticles.

### 3.4. Properties of the Coatings Produced by the Duplex Methods with a Nanoparticle Concentration of 10 g/L and 20 g/L

The optimisation focused on increasing the concentration of Al_2_O_3_ nanoparticles in the aqueous suspension to 10 and 20 g/L and extending the impregnation time to 20 and 30 min.

#### 3.4.1. The Morphology of the Coatings

**Figure 8:**
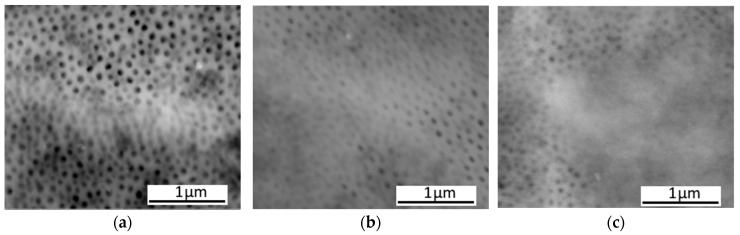
SEM-SE morphology of the coatings modified with Al_2_O_3_ nanoparticles produced by the duplex method (10 min ultrasonic impregnation) at different nanoparticle concentrations of 5 g/L (**a**), 10 g/L (**b**) and 20 g/L (**c**).

The surface morphologies of the coatings after ultrasonic impregnation in an aqueous suspension of Al_2_O_3_ nanoparticles at concentrations of 5, 10 and 20 g/L are shown in Figure 8.

SEM structural analysis shows that the particle amount on the coating surface increases with concentration. However, at 20 g/L, agglomerates are visible.

#### 3.4.2. Microhardness and Abrasion Resistance

**Figure 9:**
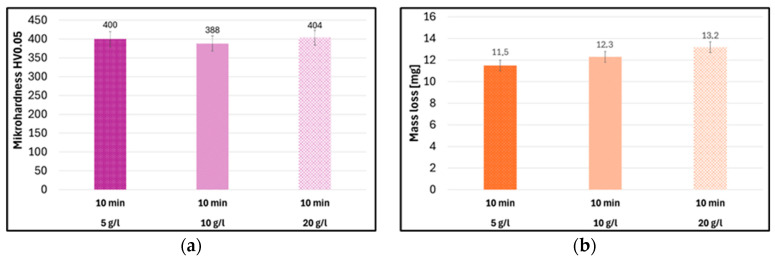
Vicker’s microhardness of coatings (**a**) and abrasion resistance (**b**) modified with Al_2_O_3_ nanoparticles produced by the duplex method (10 min ultrasonic impregnation) at different nanoparticle concentrations.

Increasing the particle concentration did not significantly increase the microhardness (Figure 9a) and led to higher mass loss in the Taber abrasion test (Figure 9b).

**Figure 10:**
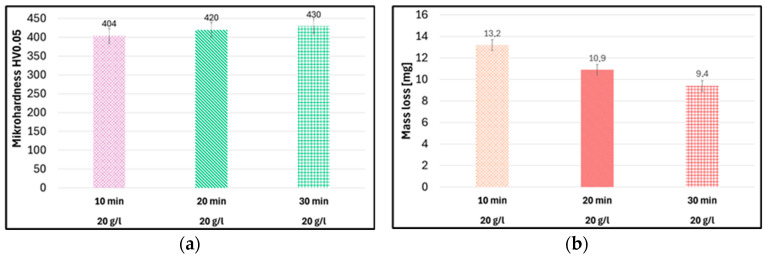
Vicker’s microhardness of coatings (**a**) and abrasion resistance (**b**) modified with 20 g/L Al_2_O_3_ nanoparticles produced by the duplex method at different impregnation times.

Increasing the impregnation time to 30 min resulted in a significant increase in microhardness (Figure 10a) and significantly reduced the mass loss, improving the modified coating’s wear resistance (Figure 10b).

#### 3.4.3. Summary of Section 3.4

To achieve a coating with improved microhardness and enhanced abrasion resistance, the most effective method involves an impregnation time of 30 min at a concentration of 20 g/L. This method results in a high particle content on the surface without the formation of agglomerates. Consequently, it leads to a significant increase in microhardness and a reduction in mass loss, ultimately providing the best wear resistance among the coatings analysed.

## 4. Discussion

One of the key challenges in working with nanoparticles is their tendency to agglomerate, which results from van der Waals interactions and interactions related to the electrostatic double layer [27]. To prevent this phenomenon, surfactants are commonly used. By adsorbing onto the surface of nanoparticles, they reduce the interfacial tension and create an electrostatic barrier, thereby stabilising the colloidal system. Traditionally, selecting a suitable surfactant required numerous laboratory trials. Today, a modern and more efficient approach involves the use of zeta potential measurements, which allow for the assessment of the stability of the colloidal system and the effectiveness of the applied surfactant. The pH is one of the most important factors affecting the value of the zeta potential, particularly in aqueous solutions where the main ions are H^+^ and OH^−^ [28]. Therefore, the studies were conducted over a wide pH range of 2–6, changing the pH in increments of one unit. Among the three analysed surfactants (SDBS, DSS and LSA), selected based on a review of the relevant literature and the author’s previous experience with nanoparticles, DSS was identified as the surfactant most effective in reducing agglomeration. Zeta potential analysis showed that, at a pH 2, the addition of the sodium salt of 2-ethylhexyl sulfosuccinate (DSS) stabilised all types of ceramic particles, at both 0 °C and 25 °C. These parameters made it possible to obtain zeta potential values corresponding to at least moderate dispersion stability (for Al_2_O_3_) and, in the case of PTFE, high dispersion stability (Table 2). A pH of 2 is the standard pH value for the anodising bath used by Łukasiewicz-IMN. When combined with the DSS surfactant, it enables the formation of a stable dispersion. Various surfactants are used in the literature for coating modification; however, detailed analyses of the zeta potential are rare. Chen et al. [29] used Triton X-100, Mohhamadi et al. [30] used SDBS, Ghalmi et al. [9] and Menini et al. [31] ZONYL^®^FS300, while Zhang et al. [4] applied SDBS and FC-10. A few researchers, such as Escobar et al. [8], reported zeta potential values (for PTFE suspensions: −50 and −45 mV) and Curioni et al. [32] recorded −26.8 mV for Ag nanoparticles in a Keronite electrolyte. Nowak et al. [33] demonstrated that a stable dispersion (−30 to −60 mV) and the effective incorporation of SiC particles into a nickel coating were achieved only with mixtures of LSA + DSS and LSA + SDS surfactants. Only a few studies report zeta potential values, as most research focuses primarily on the effects of coating modification (e.g., improved hardness or uniformity) rather than on the detailed mechanisms of particle suspension stabilisation in the electrolyte. However, it is the zeta potential that determines the stability of the suspension and the uniform deposition of particles; therefore, its measurement allows for the better selection of surfactants and the optimisation of the process, leading to improved coating uniformity and properties.

A major scientific challenge in producing nanoparticle-modified hard anodic oxide coatings lies in selecting a production method that enables uniform and maximised nanoparticle incorporation within the coating structure, thereby enhancing its mechanical and tribological properties. The coatings on the EN AW-5754 aluminium alloy were fabricated using two approaches: a direct method, involving single-step particle incorporation during anodising, and a two-step duplex method, comprising TAPT coating production followed by ultrasonic impregnation of nanoparticles. The use of two methods made it possible to select the more advantageous one, ensuring the penetration of nanoparticles into the coating structure and their uniform distribution within the TAPT structure.

On the surfaces of all coatings produced by both the direct and duplex methods, regions with closed pores (visible as brighter areas) were observed (Figure 2). Chemical composition analysis in these regions confirmed the presence of elements characteristic for the incorporated modifying particles: Al and O (Al_2_O_3_), F and C (PTFE), and Ca (CaCO_3_). However, due to the nanometric size and low concentration of the incorporated particles, the EDS detection of some elements, particularly light ones such as F, Ca and N, was considerably limited. In the case of coatings modified with Al_2_O_3_, particle identification within the microstructure was especially challenging because their chemical composition was identical to that of the oxide matrix.

Hard anodic oxide coatings are of industrial importance, and their evaluation requires compliance with ISO 10074 [26] and Qualanod [34] specifications, defining minimum microhardness (≥300 HV0.05) and Taber mass loss (≤25 mg). This study aimed to develop coatings that not only meet these requirements but also exhibit an at least 20% higher microhardness and abrasion resistance than the commercial coating produced at the Łukasiewicz-IMN anodising facility. For CaCO_3_ particles, no improvement in the microhardness or abrasion resistance was observed regardless of the modification method. In the direct method, the obtained values (432 HV0.05; 11.3 mg) were comparable to the unmodified coating (444 HV0.05; 10.6 mg) and superior to the commercial one, whereas the duplex method led to a pronounced deterioration (298 HV0.05; 19.3 mg). A similar trend was found for PTFE particles: direct modification did not affect microhardness relative to the unmodified coating (445 HV0.05) but reduced abrasion resistance (14.4 mg), while the duplex method further degraded both properties (324 HV0.05; 13.8 mg).

The reduction in microhardness and abrasion resistance in PTFE-modified coatings may stem primarily from the poor adhesion of PTFE particles to the oxide structure, as reported by Kwolek et al. [35], who observed their early detachment during abrasion and the resulting increase in mass loss. Moreover, ultrasonic impregnation, as demonstrated by Wang et al. [36], further decreases microhardness from ~370 to ~310 HV0.025, whereas subsequent heat treatment allows it to increase to ~400 HV0.025. Data from the literature also indicate a wide variability in the microhardness of PTFE-containing coatings, which is strongly dependent on the processing parameters and substrate type: Liu et al. [37] reported values of 400–480 HV for composite coatings on a 6063 alloy, while Zhang et al. [4] obtained 250–350 HV0.05 for coatings on 2xxx-series alloys modified by electrophoretic deposition. This variability confirms that the mechanical and tribological behaviour of coatings containing soft PTFE particles is strongly governed by the particle incorporation mechanism and the stability of their anchoring within the oxide structure. Comparisons among studies remain challenging due to the large number of processing parameters influencing the final coating properties.

Soft PTFE and CaCO_3_ particles primarily act as solid lubricants. Their presence promotes a reduction in shear stresses in the friction zone through the formation of a transfer film; however, their weak adhesion to the oxide structure may lead to early particle detachment during abrasive wear, resulting in a decrease in microhardness and an increase in the coating’s mass loss [36]. Data from the literature indicate considerable variability in the mechanical properties of such composite coatings, which is strongly dependent on the particle incorporation method, the type of aluminium alloy and the processing parameters [17,37].

A different interaction mechanism is exhibited by hard Al_2_O_3_ particles which, due to their hardness, are able to effectively transfer applied loads and restrict local plastic deformation of the surface layer. Under friction conditions, these particles may be progressively embedded into the coating structure and become anchored within its near-surface region. This process leads to stabilisation of the contact layer and an enhancement of abrasive wear resistance by limiting the direct interaction between the counter body and the coating matrix [7].

For coatings modified with Al_2_O_3_ particles, a beneficial increase in microhardness by approximately 48% relative to the commercial coating was achieved only when the direct method was applied; however, no improvement in abrasion resistance was observed. The Al_2_O_3_ particles were deposited predominantly within the surface layer, as confirmed by SEM (Figure 3a,d) analyses. Such localisation enhances the microhardness measured at the surface but does not translate into improved tribological performance, which depends not only on surface properties but also on the structural uniformity and integrity of the oxide layer.

Reports in the literature indicate that incorporation of hard particles, such as Al_2_O_3,_ into anodic oxide coatings can improve functional performance, although the effect is often limited and strongly dependent on processing conditions. Remesova et al. [38] observed only a minor increase in hardness (up to 510 HV0.05, ~4% above the unmodified coating) for coatings produced on a 1xxx-series Al alloy and modified with a mixture of Al_2_O_3_ and PTFE particles. Similarly, Chen et al. [17] reported a microhardness of 435 HV0.05 for Al_2_O_3_-modified coatings on a 2xxx-series alloy, corresponding to an improvement of approximately 10% compared with the unmodified coating. These results indicate that the effectiveness of incorporating hard particles is governed primarily by their distribution and degree of integration within the oxide structure.

Based on the conducted investigations, the duplex method, combined with the use of Al_2_O_3_ nanoparticles, was identified as the most effective approach for modifying hard anodic oxide coatings. This process was subsequently optimised by analysing the influence of nanoparticle concentration and processing time, as both factors have a significant impact on the effectiveness of ultrasonic impregnation.

Increasing particle concentration in suspension led to higher mass loss in the Taber abrasion test. However, extending impregnation to 30 min at 20 g/L significantly reduced mass loss, improving the modified coating’s wear resistance compared to the commercial coating. Increasing the particle concentration did not significantly increase the microhardness, while increasing the impregnation time to 30 min resulted in a significant increase in microhardness compared to the commercial coating.

Similar relationships were reported by Li et al. [39], who demonstrated that higher nanoparticle concentrations in the electrolyte promote particle agglomeration and increase the brittleness of the coating. Such agglomerates act as local stress concentrators and facilitate the initiation of microcracks, which, under abrasive loading accelerate the detachment of coating fragments. This phenomenon is consistent with the observations of Shi et al. [40], who also found that excessive particle incorporation does not enhance the wear resistance of PEO coatings and may even reduce it.

Extending the impregnation time to 30 min at 20 g/L significantly reduced the mass loss, indicating more effective and uniform pore filling. Similar effects, and improved pore filling and enhanced structural integrity, were also reported by Korzekwa et al. [6].

Increasing the particle concentration in the suspension did not significantly affect the microhardness, which can be explained by the fact that a higher number of particles does not guarantee their effective incorporation into the coating, especially when agglomeration occurs, as is discussed by Li et al. [39]. A substantial increase in microhardness was achieved only after extending the impregnation time, suggesting that uniform particle distribution and better pore filling (rather than particle quantity) are responsible for improved mechanical performance. This mechanism is consistent with the conclusions of Jia et al. [41], who stated that the reinforcing effect of nanoparticles depends primarily on their dispersion and integration with the matrix rather than on their concentration alone.

## 5. Conclusions

- Anodic oxide coatings modified with Al_2_O_3_, PTFE and CaCO_3_ nanoparticles were successfully produced using both the direct and duplex methods. However, the number of incorporated particles was small, and they were mainly located on the coating surface.
- Using the duplex method with optimised process parameters, it was possible to produce Al_2_O_3_-modified anodic oxide coatings whose properties significantly exceeded both the requirements specified by applicable standards and those of the commercially produced coating. Extending the impregnation time to 30 min and increasing the nanoparticle concentration to 20 g/L resulted in a 28% increase in microhardness and an approximately 32% improvement in abrasion resistance compared to the commercial coating.
- The modification of the coatings by the direct method, with the addition of hard Al_2_O_3_ particles, improved the mechanical properties but did not improve the abrasive wear resistance.
- The use of soft PTFE and CaCO_3_ particles as modifiers did not improve the coatings’ properties.

## Figures and Tables

**Table 1:** Chemical composition of EN-AW 5754.

Element	Al	Si	Fe	Cu	Mn	Cr	Ni	Zn	Pb	Sn	Ti
[wt. %]	Rest	0.390	0.306	0.065	0.286	2.72	0.017	0.081	0.0031	0.0006	0.028

**Table 2:** Zeta potential [mV] for nanoparticle suspensions with different surfactants at pH 2, at 0 °C.

Types of Nanoparticles	Surfactant			
	Without	DSS	LSA	SDBS
Al_2_O_3_	−8.1	−36.5	−12.2	−27.5
PTFE	−6.1	−88.8	−29.4	−92.6
CaCO_3_	−0.7	−63.2	−4.8	−49.5

**Table 3:** Electrochemical test results for hard anodic oxide coatings modified with nanopowders.

Type of Coating	E_corr_ [mV]	I_corr_ [A/dm^2^]	Rp [Ω]
Without particles—commercial	−783	2.34 × 10^−8^	1.28 × 10^6^
Without particles	−842	1.00 × 10^−7^	1.40 × 10^5^
B/Al_2_O_3_	−965	5.44 × 10^−10^	2.70 × 10^7^
B/PTFE	5	5.44 × 10^−10^	2.70 × 10^7^
B/CaCO_3_	−599	9.00 × 10^−10^	1.60 × 10^7^
D/Al_2_O_3_	−623	1.80 × 10^−6^	1.30 × 10^4^
D/PTFE	−109	3.30 × 10^−8^	4.60 × 10^5^
D/CaCO_3_	−923	3.10 ×·10^−8^	9.50 ×·10^4^

## Data Availability

The original contributions presented in this study are included in the article/Supplementary Material. Further inquiries can be directed to the corresponding authors.

## Supplementary Materials

The following supporting information can be downloaded at: https://www.mdpi.com/article/10.3390/ma19020378/s1, Figure S1: EDS analysis of the cross-sections of the coatings produced by the direct method.
