# Supplementary material for: The Microstructure and Properties of Hard Anodic Oxide Coatings on 5754 Aluminium Alloy Modified with Al2O3, PTFE and CaCO3 Nanoparticles
Source: Materials (Basel). 2026 Jan 17;19(2):378. doi: 10.3390/ma19020378 (PMC12842711; doi:10.3390/ma19020378)
Supplement: Supplementary file 1 [file materials-19-00378-s001.zip › materials-4098468-supplementary.pdf]

Element	% wt.	% at.
O	50.6	63.3
Mg	1.5	1.2
Al	47.9	35.5
**Total**	100	100

(**a**)

Element	% wt.	% at.
O	52.5	63,5
Mg	1.4	1,1
Al	45.9	35,2
F	1.1	1,2
**Total**	100	100

(**b**)

Element	% wt.	% at.
O	51.8	65.4
Mg	1.7	1.4
Al	43.4	32.5
Ca	0.2	0.1
Pt	2.4	0.2
**Total**	100	100

(**c**)

Element	% wt.	% at.
O	53.8	66.2
Mg	1.3	1.1
Al	44.8	32.7
**Total**	100	100

(**d**)

Element	% wt.	% at.
O	49.0	53.7
Mg	1.2	0.8
Al	33.3	21.6
C	16.1	23.5
F	0.4	0.4
**Total**	100	100

(**e**)

Element	% wt.	% at.
O	48.8	61.7
Mg	1.3	1.1
Al	48.9	36.7
Ca	1.1	0.5
**Total**	100	100

(**f**)

Figure S1. EDS analysis of the cross-sections of the coatings produced by the direct method: (**a**) Al_2O_3, (**b**) PTFE, and (**c**) $CaCO_3$; and by the duplex method: (**d**) Al_2O_3, (**e**) PTFE, and (**f**) $CaCO_3$
